# Benign Multicystic Peritoneal Mesothelioma Presenting as a Colonic Mass

**DOI:** 10.7759/cureus.15540

**Published:** 2021-06-09

**Authors:** Kevin Pierre, Noah F Gomez, Shaoxu Bing, Carolina E Garcia, Brian G Dalton

**Affiliations:** 1 General Surgery, University of Florida College of Medicine – Jacksonville, Jacksonville, USA

**Keywords:** diverticulitis, abdominal mass, abdominal fluid collection, benign multicystic peritoneal mesothelioma, general surgery

## Abstract

Benign multicystic peritoneal mesothelioma (BMPM) is a rare neoplasm of the abdominal mesothelium (i.e., peritoneum, mesentery, and omentum). We present the case of a 74-year-old male who presented with a right paracolic gutter fluid collection and cystic mass. The patient underwent diagnostic laparoscopy with resection of the mass. The final pathology revealed BMPM. The pathogenesis may have been related to longstanding diverticular disease, which could prove to be an underrecognized risk factor for the development of BMPM. Therefore, this case suggests a broadened differential diagnosis to include BMPM in specific cases of pre-operatively diagnosed colonic masses. The patient is disease-free 11 months post-operatively.

## Introduction

Benign multicystic peritoneal mesothelioma (BMPM) is a loculated neoplasm arising from the mesothelial lining of the abdominal cavity [1]. BMPM is a rare disease (annual incidence of 0.15/100,000 [2]) with <200 reported cases [3] since it was first described in 1928 [4], although this number may be an underestimate considering it is a benign condition. Surgeons may not be familiar with this uncommon disease due to the scant number of cases. Therefore, we present the case of a 74-year-old male with BMPM initially diagnosed as a colonic mass.

## Case presentation

**Figure 1:**
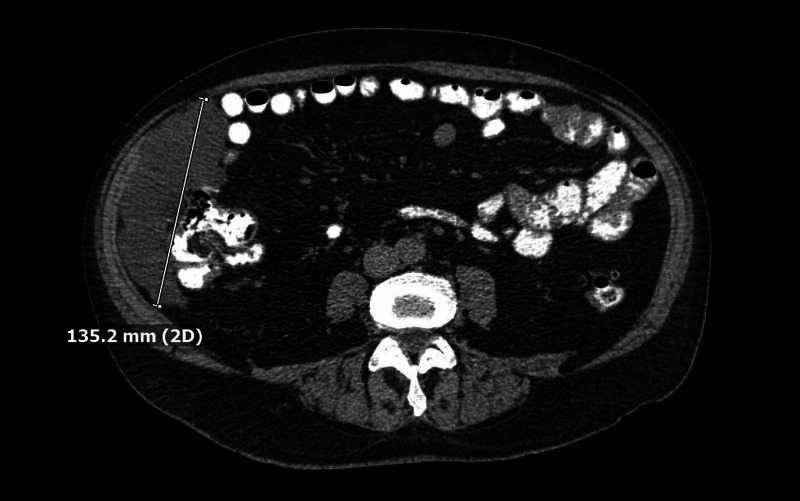
Abdominal CT (November 2018). CT of the abdomen showing a 13.5 cm right lower quadrant (RLQ) paracolic gutter fluid collection.

A 74-year-old male with a past medical history of diverticulitis presented to the clinic in November 2018 for evaluation of a recurrent 13.5 cm right lower quadrant (RLQ) paracolic gutter fluid collection (Figure 1). The patient was first diagnosed with an intraabdominal fluid collection in 2017, status post CT-guided interventional radiology (IR) drainage, and positron emission tomography CT scan, which were negative for malignancy. Routine colonoscopy in July 2018 was significant for small-mouthed diverticula in the sigmoid colon and non-bleeding internal hemorrhoids. Thereafter, the intraabdominal fluid collection re-accumulated and subsequent CT-guided IR drainage showed no evidence of malignancy.

**Figure 2:**
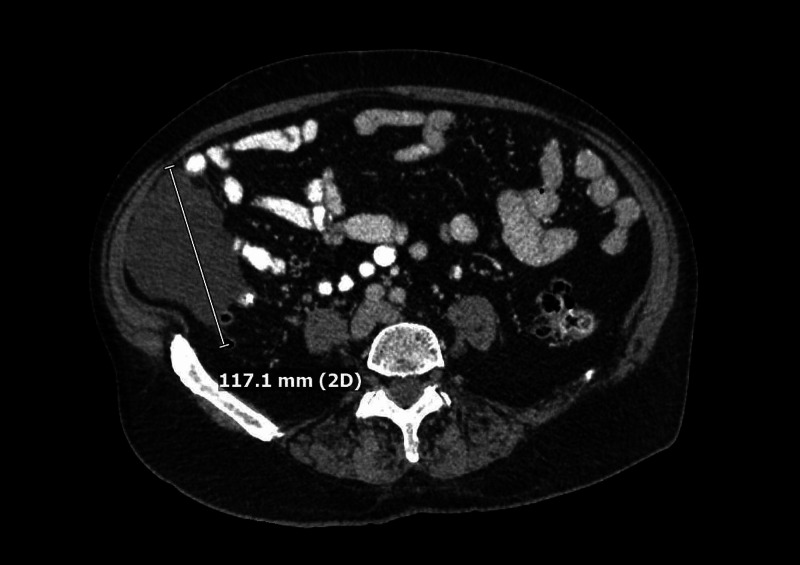
Abdominal CT (August 2019). CT of the abdomen showing a right paracolic gutter lobular fluid-attenuated structure with features of a cystic lymphangioma measuring 11.7 cm.

Follow-up CT in August 2019 was significant for severe colonic diverticulosis and a right paracolic gutter lobular fluid-attenuated structure with features of a cystic lymphangioma that measured 11.7 cm (Figure 2). The patient opted for continued observation and repeat imaging.

**Figure 3:**
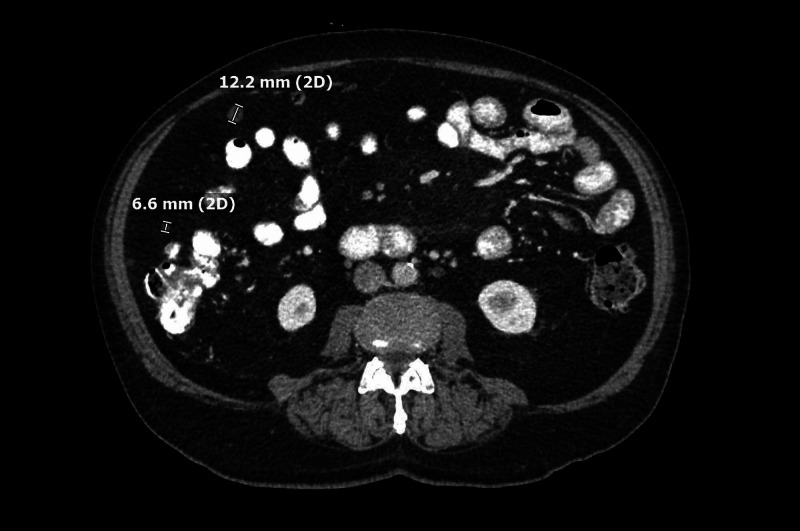
Abdominal CT (January 2020). CT of the abdomen showing numerous scattered mesenteric and peritoneal nodules.

Repeat imaging in January 2020 showed a similar mass, in addition to numerous scattered mesenteric and peritoneal nodules within the abdomen and pelvis concerning a peritoneal neoplasm (Figure 3).

In February 2020, the patient underwent diagnostic laparoscopy with excision of the cystic mass (Video 1).

**Video 1:** Laparascopic resection of benign multicystic mesothelioma.

**Figure 4:**
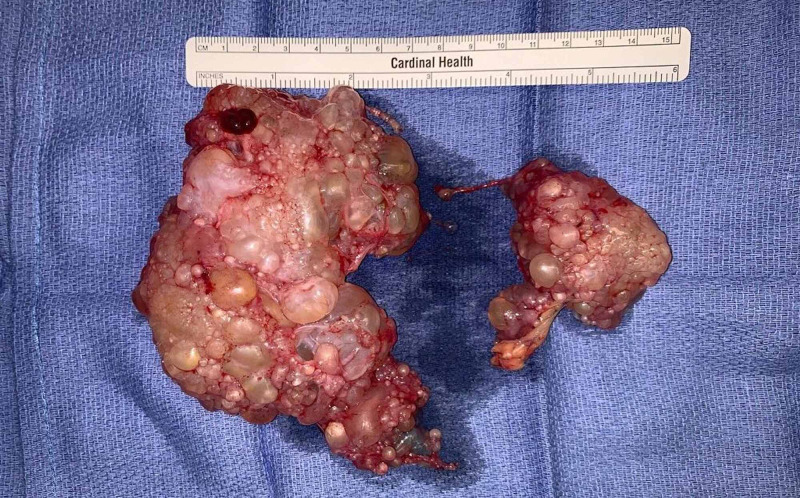
Gross surgical specimens. The right lower quadrant (RLQ) mass measured approximately 13 cm and the left upper quadrant (LUQ) mass measured approximately 5 cm.

In the RLQ, a complex 13 cm cystic mass appeared to be arising from the omentum. A similar 5 cm cystic mass was identified in the left upper quadrant (LUQ) (Figure 4).

**Figure 5:**
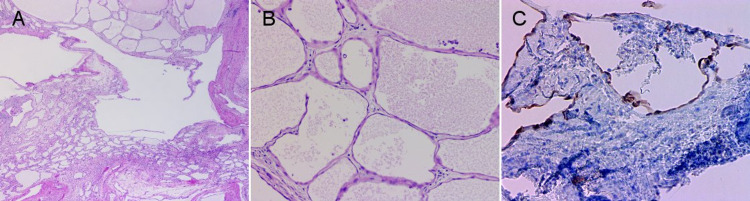
Photomicrographs of H&E and immunohistochemical-stained specimens. Photomicrographs of benign multicystic peritoneal mesothelioma (BMPM) showing multiple cysts lined by a single layer of bland, flat to the cuboidal epithelium (H&E stain; 20X (A), 200X (B)) and cysts lined with calretinin-positive cells (200X (C)).

Final pathology revealed benign multicystic mesothelioma with calretinin-positive immunohistochemical staining (Figure 5). The patient was discharged postoperative day 1, recovered well, and is now 11 months post-operative without evidence of recurrent disease.

## Discussion

BMPM is 4-5 times more common in females and typically presents in premenopausal women (<30 years old) with risk factors like endometriosis, pelvic inflammatory disease, and a history of abdominal surgery [1]. BMPM can arise from any serosal-lined surface in the peritoneal cavity [5], including the ovaries, uterus, and rarely, the omentum [6]. The proliferation of the serosal epithelium results in non-specific symptoms such as abdominal fullness, abdominal pain, and constipation, secondary to tumor mass effect [7]. The pathogenesis of BMPM is undetermined but may result from serosal inflammation, among other proposed etiologies [8]. Treatment relies primarily on surgical resection but may also include tamoxifen [9], gonadotropin-releasing hormone (GnRH) agonists [10], rapamycin [11], and hyperthermic intraperitoneal chemotherapy [12]. Although the recurrence rate is nearly 50% in women and 33% in men [3], long-term survival is favorable [1].

Our case contributes to the rare instances of BMPM in men [13] and the uncommon cases of BMPM in patients with the diverticular disease [14,15]. It is plausible that serosal inflammation secondary to diverticulitis provided a reactive milieu for the development of BMPM, considering that our patient had no other risk factors. Therefore, we suggest that BMPM be a diagnostic consideration in patients with a history of diverticular disease presenting with an imaging-detected colonic mass after colon cancer is ruled out. Although the leading differential diagnosis for a large peritoneal mass would be malignant mesothelioma, which closely resembles BMPM on microscopy, the lack of tissue invasion favors the diagnosis of BMPM. The incidental finding of a second BMPM in the LUQ underscores the limitations of imaging studies in localizing BMPM pre-operatively. Therefore, a thorough evaluation of the entire omentum for additional masses and nodules should be performed in all patients with BMPM. While there are no established surveillance guidelines, a recommendation of repeat CT every three months for a year, followed by annual CT scans for the next five years has been published [3].

## Conclusions

While BMPM is a rare neoplasm, especially in males, it should be considered in the differential diagnosis of a colonic mass in patients with a benign recurrent abdominal fluid collection in the setting of diverticular disease and normal colonoscopy. Our case highlights the need for further identification of BMPM risk factors, which may include GI disease that extends to the colonic serosa (e.g., diverticulitis). While our patient is currently disease-free, there is a high likelihood that BMPM may reoccur. Therefore, further investigation into the etiology of BMPM may elucidate novel post-surgical medical treatments for preventing disease recurrence.

## References

[1] Chand MT, Edens J, Lin T, Anderson I, Berri R (2020). Benign multicystic peritoneal mesothelioma: literature review and update. Autops Case Rep.

[2] Snyder JA, Carman R Jr, Aggon AA, Cardinale JP (2011). Benign multicystic peritoneal mesothelioma: a rare case presenting as pneumoperitoneum and pneumotosis intestinalis. J Gastrointest Oncol.

[3] Khurram MS, Shaikh H, Khan U (2017). Benign multicystic peritoneal mesothelioma: a rare condition in an uncommon gender. Case Rep Pathol.

[4] Canbay E, Ishibashi H, Sako S, Kitai T, Nishino E, Yonemura Y (2013). Late recurrence of benign multicystic peritoneal mesothelioma complicated with an incisional hernia. Case Rep Surg.

[5] Pitta X, Andreadis E, Ekonomou A (2010). Benign multicystic peritoneal mesothelioma: a case report. J Med Case Rep.

[6] Alhatem A, Marcus J, Heller DS (2019). Multicystic mesothelioma of the omentum presenting as an incidental finding. Int J Surg Pathol.

[7] Momeni M, Pereira E, Grigoryan G, Zakashansky K (2014). Multicystic benign cystic mesothelioma presenting as a pelvic mass. Case Rep Obstet Gynecol.

[8] Takemoto S, Kawano R, Honda K, Nakazono A, Shimamatsu K (2012). Benign multicystic peritoneal mesothelioma mimicking recurrence of an ovarian borderline tumor: a case report. J Med Case Rep.

[9] Letterie GS, Yon JL (1998). The antiestrogen tamoxifen in the treatment of recurrent benign cystic mesothelioma. Gynecol Oncol.

[10] Letterie GS, Yon JL (1995). Use of a long-acting GnRH agonist for benign cystic mesothelioma. Obstet Gynecol.

[11] Stallone G, Infante B, Cormio L, Macarini L, Grandaliano G (2017). Rapamycin treatment for benign multicystic peritoneal mesothelioma: a rare disease with a difficult management. Am J Case Rep.

[12] Gussago S, Spina P, Guerra A (2019). Benign multicystic peritoneal mesothelioma (BMPM) as a rare cause of abdominal pain in a young male: case report and review of the literature. J Surg Case Rep.

[13] Canu GL, Medas F, Columbano G, Gordini L, Saba L, Erdas E, Calò PG (2018). Benign multicystic peritoneal mesothelioma in a male patient with previous wilms' tumor: a case report and review of the literature. Case Rep Surg.

[14] Bansal A, Zakhour HD (2006). Benign mesothelioma of the appendix: an incidental finding in a case of sigmoid diverticular disease. J Clin Pathol.

[15] Tangjitgamol S, Erlichman J, Northrup H, Malpica A, Wang X, Lee E, Kavanagh JJ (2005). Benign multicystic peritoneal mesothelioma: cases reports in the family with diverticulosis and literature review. Int J Gynecol Cancer.

